# *Anncaliia algerae* Microsporidiosis Diagnosed by Metagenomic Next-Generation Sequencing, China

**DOI:** 10.3201/eid2807.212315

**Published:** 2022-07

**Authors:** Chen Liu, Qin Chen, Ping Fu, Yun-Ying Shi

**Affiliations:** West China Hospital of Sichuan University, Chengdu, China (C. Liu, Q. Chen, P. Fu, Y.-Y. Shi);; Chengdu 363 Hospital of Southwest Medical University, Chengdu (Q. Chen)

**Keywords:** fungi, parasites, Anncaliia algerae, metagenomics, next-generation sequencing, microsporidiosis, myositis, immunocompromised, kidney transplantation, transplant recipients, China

## Abstract

We report a case of *Anncaliia algerae* microsporidia infection in an immunosuppressed kidney transplant recipient in China. Light microscopy and transmission electron microscopy initially failed to identify *A. algerae*, which eventually was detected by metagenomic next-generation sequencing. Our case highlights the supporting role of metagenomic sequencing in early identification of uncommon pathogens.

*Anncaliia algerae* is an uncommon, yet emerging microsporidian parasitic pathogen that can affect immunocompromised patients and cause fatal myositis (*1*,*2*). We report a case of *A. algerae* microsporidiosis, which was initially missed by conventional light microscopy (LM) and subsequent transmission electron microscopy (TEM) of biopsied muscle but eventually identified by metagenomic next-generation sequencing (mNGS).

## The Study

In March 2021, a 45-year-old male kidney transplant recipient in China was admitted to the hospital for a 2-month history of muscle pain. He was receiving prednisone, tacrolimus, and mycophenolate mofetil for maintenance immunosuppression. The patient did not have respiratory symptoms at admission. Physical examination showed low fever and tenderness and generalized weakness in all 4 limbs. Laboratory investigations revealed serum creatine kinase level within reference range but low CD4+ T lymphocyte count (45 cells/µL; reference range 471–1,220 cells/µL). Serum cytomegalovirus DNA was 1.64 × 10^2^ copies/mL. Results of tests for heavy metals, parasites, and myositis-specific autoantibodies were negative.

**Figure 1:**
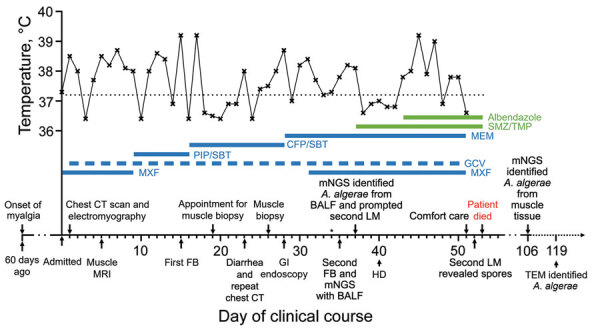
Clinical course of a 45-year-old patient with *Anncaliia algerae* microsporidia infection, China. The upper section of the graph shows the body-temperature curve (black line); dotted black line indicates 37.2°, the upper limit of normal body temperature. Thick blue and green lines indicate medications administered; dashed thick blue line indicates a dosing frequency of every other day. Major events during the patient’s course are indicated by arrows on the x-axis. Asterisk on day 34 denotes the initial light microscopy, which failed to detect *A. algerae* spores. BALF, bronchoalveolar lavage fluid; CFP/SBT, cefoperazone/sulbactum; CT, computed tomography; FB, fiberoptic bronchoscopy; GCV, ganciclovir; GI, gastrointestinal; HD, hemodialysis; LM, light microscopy; MEM, meropenem; mNGS, metagenomic next-generation sequencing; MRI, magnetic resonance imaging; MXF, moxifloxacin; PIP/SBT, piperacillin/sulbactum; SMZ/TMP, sulfamethoxazole/trimethoprim; TEM, transmission electron microscopy.

The patient was febrile (37.3°C) at admission. Although immunosuppressant drugs were tapered dramatically, and broad-spectrum antimicrobial drugs and ganciclovir were added, the patient remained febrile (Figure 1). Chest computed tomography (CT) imaging showed patchy irregular ground-glass opacity in the left upper lung lobe. Electromyography testing showed myogenic damage in the biceps brachii muscle. Magnetic resonance imaging of lower extremities revealed swollen soft tissue. Bronchoalveolar lavage (BAL) testing was negative for bacteria, fungi, and *Pneumocystis jirovecii* DNA.

The patient’s myalgia and weakness worsened, his serum creatine kinase level increased (Appendix Figure 1), and watery diarrhea developed. Stool microscopy, gastroduodenoscopy, and colonoscopy revealed no specific abnormalities; repeated chest CT scans showed increased inflammatory exudation and bilateral pleural effusion.

**Figure 2:**
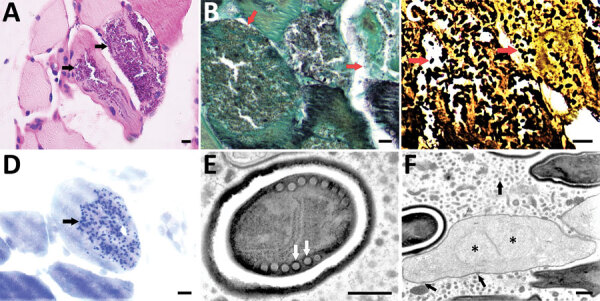
Light microscopy and transmission electron microscopy of left biceps branchii muscle biopsy tissue from a 45-year-old man with microsporidiosis caused by *Anncaliia algerae*, China. A–D) Light microscopy using different stains. A) Periodic acid-Schiff stain. Scale bar indicates 10 µm. Original magnification ×50. B) Gomori methenamine silver stain. Scale bar indicates 10 µm. Original magnification ×63. C) Warthin-Starry stain. Scale bar indicates 10 µm. Original magnification ×63. D) Toluidine blue stain. Scale bar indicates 10 µm. Arrows indicate myocytes replaced by aggregates of 2–3 µm ovoid organisms. Original magnification ×63. E, F) Transmission electron microscopy showing *Anncaliia*-like microsporidia. Scale bars indicate 500 nm. E) A mature spore with electron-dense exospore, electron-lucent endospore, and a single row of 6 to 8 polar tubule coils (arrows). Original magnification ×8,000. F) Proliferating form of microsporidia showing diplokaryotic nuclei (stars) with vesiculotubular structures extending from the meront cell membrane and aggregating in the host cell cytoplasm (arrows). Original magnification ×3,000.

No specific findings were reported from the initial LM of the left biceps brachii biopsy specimen except for degradation and necrosis of myofibers. We performed a second fiberoptic bronchoscopy and sent BAL fluid for untargeted mNGS via NextSeq 550 (Illumina, https://www.illumina.com), which revealed *P. jirovecii* (142 sequence reads) and *A. algerae* (127 sequence reads) within 48 hours of receiving the specimen (Appendix Table 1, Figure 2, panel A). 

Because the previous biopsy results were negative and we were unfamiliar with *A. algerae* microsporidia, we performed a literature review and then reviewed the initial muscle biopsy again. We considered the possibility of a combined infection of *P. jirovecii* and *A. algerae*, and we consulted an infectious disease specialist who suggested adding oral sulfamethoxazole/trimethoprim (SMZ/TMP; 1,600/320 mg 3×/d), which might be effective against both pathogens. After SMZ/TMP treatment, the patient’s temperature returned to normal for 5 successive days before climbing to 37.8°C on day 43 of admission; we added oral albendazole (400 mg 2×/d) (Figure 1), according to published cases (*1*,*3*,*4*). 

However, the patient’s condition continued to deteriorate. On day 51, he decided on comfort care and died 2 days later (Figure 1). On day 52, one day before the patient died, we discovered multiple oval organisms measuring 2–3 µm in scattered clusters under LM in the muscle biopsy sample (Figure 2, panels A–D). After the patient died, we performed mNGS using muscle tissue from the previous biopsy, which yielded 65,311 sequence reads mapped to *A. algerae* (Appendix Table 2, Figure 2, panel B). *A. algerae* was confirmed by subsequent PCR testing on muscle tissue, but PCR testing of the remaining BAL specimen yielded no findings because not enough fluid was available in the sample after previous examinations. Eventually, we identified *A. algerae* via TEM in the third sample section (Figure 1; Figure 2, panels E, F). We deposited the *A. algerae* sequences into the National Center for Biotechnology Information Sequence Read Archive (accession nos. SRR18339014 for the BAL sample, SRR18339013 for the muscle sample).

## Conclusions

*A. algerae* is a microsporidial species that has been reported to cause human infections since 1999 (*5*). Of 12 reported cases of human *A. algerae* infection (*1*–*11*), 11 were among immunocompromised patients (Table). Thus, immunodeficiency, as in this patient, appears to be a critical risk factor for *A. algerae* infection. Although the modes of *A. algerae* transmission to humans remain uncertain, waterborne transmission, either through ingestion of or exposure to spore-contaminated water, has been postulated as the most likely route (*2*,*4*,*6*). This patient lived near ditches in a rural area of the warm and humid Sichuan Basin and was readily exposed to waters possibly contaminated by *A. algerae* spores.

**Table:** Clinical characteristics of 12 previously reported cases of human *Anncaliia algerae* microsporidia infection*

Case reports	Age, y/sex	Immunocompromised/underlying conditions	Related symptoms	Positive biopsy sample sites	Treatment	Outcome
Watts et al. 2014 (*1*)	67/M	Y/RA	Myalgias	Vastus lateralis	Albendazole	Survived
	66/M	Y/RA	Myalgias	Vastus lateralis	NG	Died
Coyle et al. 2004 (*2*)	57/F	Y/RA	Myalgias	Quadriceps femoris	Albendazole	Died
Boileau et al. 2016 (*3*)	49/M	Y/CLL	Myalgias	Vastus lateralis	Albendazole and fumagillin	Survived
Sutrave et al. 2018 (*4*)	66/M	Y/GVHD	Myalgias	Vastus lateralis	Albendazole	Survived
Visvesvara et al. 1999 (*5*)	67/M	N/N	Eye discomfort	Cornea	Albendazole and fumagillin	Survived
Ziad et al. 2021 (*6*)	55/M	Y/psoriatic arthritis	Myalgias	Vastus lateralis, intercostal muscle, and tongue	Albendazole	Died
Visvesvara et al. 2005 (*7*)	11/M	Y/ALL	Skin lesions	Skin	NA	NA
Cali et al. 2010 (*8*)	69/M	Y/CLL	Hoarseness	False vocal cord	Albendazole	Died
Field et al. 2012 (*9*)	49/F	Y/lung transplant	Myalgias	Deltoid and tongue	NG	Died
Chacko et al. 2013 (*10*)	56/M	Y/kidney transplant	Myalgias	Deltoid	Albendazole	Died
Anderson et al. 2019 (*11*)	60/M	Y/kidney and pancreas transplant	Skin lesions	Lower extremity, finger, tongue, urine, and sputum	Albendazole	Died

*ALL, acute lymphoblastic leukemia; CLL, chronic lymphocytic leukemia; GVHD, graft-versus-host disease; NA, data not available; NG, treatment for *A. algerae* was not given because the patient was undiagnosed before death; RA, rheumatoid arthritis.

*A. algerae* infection in humans primarily manifests as myositis (*1*–*11*), and in reports we reviewed, 5 (62.5%) of 8 case-patients who had *A. algerae* myositis died (Table). Because of fatality risk, early diagnosis and prompt interventions are crucial. To date, biopsy and microscopy remain the standard approaches in microsporidia identification (*12*), and the role of mNGS has yet to be confirmed.

Although LM is the fastest diagnostic tool for microsporidiosis, it has several limitations. First, LM is unable to identify the genus and species of microsporidia. Second, the actual turnaround time (5–7 days in our hospital) for LM varies among institutions, which could cause diagnostic delays. Third, the accuracy of LM diagnosis relies on laboratory conditions and microscopist experience. In addition, morphologic features of *A. algerae* spores overlap with those of other organisms, such as small yeasts, which has led to misdiagnosis under LM (*1*,*11*). Thus, familiarity with *A. algerae* spores and their appearance on histopathology preparations are crucial for rapid diagnosis. In this case, *A. algerae* spores initially were missed by the microscopist and were detected 2 weeks later during retrospective review because of the relatively long turnaround time.

TEM remains the standard technique for determining the specific microsporidia genus by identifying the ultrastructural characteristics (*12*). TEM examines a smaller area of tissue at one time but usually has a longer turnaround time than routine LM. TEM results are available in 1–2 days in some institutions, but turnaround time in our hospital takes ≈10–14 days.

As an unbiased, culture-free method capable of detecting all potential pathogens, untargeted mNGS enables identification of unexpected or unknown organisms (*13*). Compared with hypothesis-driven methods, such as PCR, shotgun mNGS is hypothesis-free, enables survey of all DNA and RNA in multiple samples en masse (*13*), and generally takes 24–48 hours to produce results. However, mNGS is unlikely to replace conventional diagnostic testing because of its limitations, such as high cost (US $522 for DNA detection and $894 for both DNA and RNA in our hospital), lack of a unified workflow, and no standard methods for interpreting results (*13*). Instead, mNGS can serve as a valuable adjunct tool in diagnosing uncommon or unexplained infections when conventional methods such as LM fail.

Albendazole and fumagillin have been used to treat *A. algerae* infections in previously reported cases (Table). We have easy access to albendazole, but no access to fumagillin. SMZ/TMP was reported to have no effect against *Enterocytozoon bieneusi* microsporidiosis (*14*), but data regarding effectiveness against *A. algerae* microsporidia were limited. Treatment was greatly delayed in this patient because of our lack of clinical experience with *A. algerae* microsporidia and the late microscopy findings. Early treatment, along with minimized immunosuppression, might be crucial for the successful management of *A. algerae* infection (*1*,*3*,*4*).

In conclusion, *A. algerae* microsporidia infection requires early diagnosis and prompt intervention. LM alone cannot identify microsporidia genus and species; thus, TEM or genomic sequencing are needed for correct diagnosis. As a sensitive, culture-independent approach, mNGS could be a promising adjunct tool for the early identification of uncommon pathogens, such as *A. algerae* and other microsporidia.

Appendix Additional information on *Anncaliia algerae* microsporidiosis diagnosed by metagenomic next-generation sequencing, China.
