# Supplementary material for: Anncaliia algerae Microsporidiosis Diagnosed by Metagenomic Next-Generation Sequencing, China
Source: Emerg Infect Dis. 2022 Jul;28(7):1466–70. doi: 10.3201/eid2807.212315 (PMC9239868; doi:10.3201/eid2807.212315)
Supplement: Appendix — Additional information on Anncaliia algerae microsporidiosis diagnosed by metagenomic next-generation sequencing, China. [file 21-2315-Techapp-s1.pdf]

Article DOI: https://doi.org/10.3201/eid2807.212315

Anncaliia algerae Microsporidiosis Diagnosed by Metagenomic Next-Generation Sequencing, China

Appendix

Appendix Methods

Literature Review

Regarding *Anncaliia algerae* human infection, we searched English databases including PubMed and Web of Science from inception to March 28, 2022. Keywords used for searching were *Anncaliia algerae*, *Brachiola algerae*, and *Nosema algerae*.

Bronchoalveolar Lavage Fluid (BALF) Detection

We performed Gram staining and acid-fast staining of smears from BALF for bacteria detection and Gram staining for fungi.

Metagenomic Next-Generation Sequencing (mNGS)

Nucleic Acid Extraction, Library Preparation, and Sequencing

DNA was extracted from bronchoalveolar lavage fluid and muscle tissue by using a QIAamp UCP Pathogen DNA Kit (QIAGEN, https://www.qiagen.com), following the manufacturer's instructions. Human DNA was removed using Benzonase (Qiagen) and Tween20 (Sigma-Aldrich, https://www.sigmaaldrich.com) (*1*). Total RNA was extracted with a QIAamp Viral RNA Kit (Qiagen) and ribosomal RNA was removed by a Ribo-Zero rRNA Removal Kit (Illumina, https://www.illumina.com). cDNA was generated by using reverse transcription PCR and dNTPs (Thermo Fisher Scientific, https://www.thermofisher.com). Libraries were

constructed for the DNA and cDNA samples by using a Nextera XT DNA Library Prep Kit (Illumina) (*2*). Library was quality assessed by Qubit dsDNA High-Sensitivity (HS) Assay Kit (Thermo Fisher Scientific) followed by High Sensitivity DNA Kit (Agilent Technologies, https://www.agilent.com) on an Agilent 2100 Bioanalyzer. Library pools were then loaded onto an Illumina Nextseq CN500 sequencer for 75 cycles of single-end sequencing to generate ≈20 million reads for each library. For negative controls, we also prepared PBMC samples with 105 cells/mL from healthy donors in parallel with each batch, using the same protocol, and sterile deionized water was extracted alongside the specimens to serve as non-template controls (NTC) (*2*,*3*).

Bioinformatics Analyses

Trimmomatic (*4*) was used to remove low quality reads, adaptor contamination, and duplicate reads, as well as those shorter than 50 bp. Low complexity reads were removed by Kcomplexity with default parameters (*5*). Human sequence data were identified and excluded by mapping to a human reference genome (hg38) by using Burrows-Wheeler Aligner software (*6*). We designed a set of criteria similar to the National Center for Biotechnology Information (NCBI) criteria for selecting representative assembly for microorganisms (bacteria, viruses, fungi, protozoa, and other multicellular eukaryotic pathogens) from the NCBI Nucleotide and Genome databases (*7*). Pathogen lists were selected according to 3 references: 1 Johns Hopkins ABX Guide (https://www.hopkinsguides.com/hopkins/index/Johns_Hopkins_ABX_Guide/Pathogens); Manual of Clinical Microbiology (*8*); and clinical case reports or research articles published in current peer-reviewed journals (*9*). The final database consisted of ≈13,000 genomes. Microbial reads were aligned to database with SNAP version 1.0β.18 (M. Zaharia, unpub. data, https://arxiv.org/abs/1111.5572). Virus-positive detection results (DNA or RNA viruses) were defined as the coverage of ≥3 non-overlapping regions on the genome. A positive detection was reported for a given species or genus if the reads per million (RPM) ratio, or RPM-r was ≥5,

where the RPM-r was defined as the RPM sample/RPMNC (i.e., the RPM corresponding to a given species or genus in the clinical sample divided by the RPM in the NC/negative control) (*2*). In addition, to minimize cross-species misalignments among closely related microorganisms, we penalized (reduced) the RPM of microorganisms sharing a genus or family designation if the species or genus appeared in non-template controls. A penalty of 5% was used for species (*10*).

PCR Testing

Primers specific to *Anncaliia algerae* (NALGF1-TCA CCA GAG CCT ATG TGC AGG; NALGR2-CTT CAT AAA AAC ATC CAT CTC) were used and amplified from a 405-bp segment of the small ribosomal subunit ribonucleic acid gene, which showed 100% identity with previous Genbank entries (accession nos. HM216911, AM422905).

References

1. Amar Y, Lagkouvardos I, Silva RL, Ishola OA, Foesel BU, Kublik S, et al. Pre-digest of unprotected DNA by Benzonase improves the representation of living skin bacteria and efficiently depletes host DNA. Microbiome. 2021;9:123. PubMed https://doi.org/10.1186/s40168-021-01067-0

2. Miller S, Naccache SN, Samayoa E, Messacar K, Arevalo S, Federman S, et al. Laboratory validation of a clinical metagenomic sequencing assay for pathogen detection in cerebrospinal fluid. Genome Res. 2019;29:831–42. PubMed https://doi.org/10.1101/gr.238170.118

3. Li H, Gao H, Meng H, Wang Q, Li S, Chen H, et al. Detection of pulmonary infectious pathogens from lung biopsy tissues by metagenomic next-generation sequencing. Front Cell Infect Microbiol. 2018;8:205. PubMed https://doi.org/10.3389/fcimb.2018.00205

4. Xu Y, Kang L, Shen Z, Li X, Wu W, Ma W, et al. Dynamics of severe acute respiratory syndrome coronavirus 2 genome variants in the feces during convalescence. J Genet Genomics. 2020;47:610–7. PubMed https://doi.org/10.1016/j.jgg.2020.10.002

5. Bolger AM, Lohse M, Usadel B. Trimmomatic: a flexible trimmer for Illumina sequence data. Bioinformatics. 2014;30:2114–20. PubMed https://doi.org/10.1093/bioinformatics/btu170

6. Li H, Durbin R. Fast and accurate short read alignment with Burrows-Wheeler transform. Bioinformatics. 2009;25:1754–60. PubMed https://doi.org/10.1093/bioinformatics/btp324

7. National Center for Biotechnology Information. Assembly anomalies and other reasons a genome assembly may be excluded from RefSeq [cited 2021 Mar 15]. https://www.ncbi.nlm.nih.gov/assembly/help/anomnotrefseq

8. Carroll KC, Pfaller MA, Landry ML, McAdam AJ, Patel R, Richter SS, et al., editors. Manual of Clinical Microbiology, 12th edition. Washington, DC: ASM Press; 2019.

9. Fiorini N, Lipman DJ, Lu Z. Towards PubMed 2.0. eLife. 2017;6:e28801. PubMed https://doi.org/10.7554/eLife.28801

10. Gu W, Deng X, Lee M, Sucu YD, Arevalo S, Stryke D, et al. Rapid pathogen detection by metagenomic next-generation sequencing of infected body fluids. Nat Med. 2021;27:115–24. PubMed https://doi.org/10.1038/s41591-020-1105-z

Appendix Table 1. Potentially pathogenic microorganisms detected by metagenomic next-generation sequencing in bronchoalveolar lavage fluid from a patient with *Anncaliia algerae* microsporidiosis, China*

	Genus			Species	
Type	Name	Relative abundance	Sequence no.	Name	Sequence no.
G+	*Enterococcus*	33.1%	25,931	*E. faecium*	22,911
G+	*Peptostreptococcus*	2.1%	1,372	*P. anaerobius*	1,348
Fungi	*Pneumocystis*	3.2%	142	*P. jirovecii*	142
	Anncaliia	2.4%	127	*A. algerae*	127
	Candida	0.1%	9	*C. albicans*	6

*G+, gram positive bacteria.

Appendix Table 2. Potentially pathogenic microorganism(s) detected by metagenomic next-generation sequencing in muscle tissue from a patient with *Anncaliia algerae* microsporidiosis, China*

	Genus			Species	
Type	Name	Relative abundance	Sequence no.	Name	Sequence no.
Fungus	*Anncaliia*	99.9%	65,311	*Anncaliia algerae*	65,311

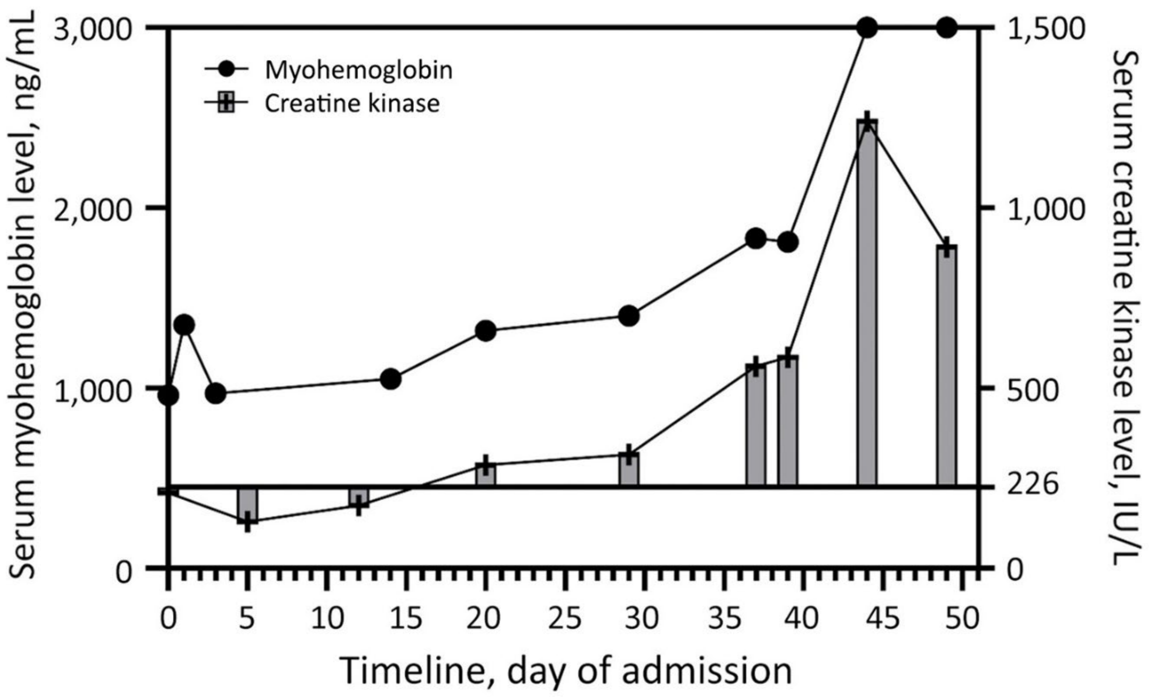

Appendix Figure 1. Trends of serum myohemoglobin (reference range 28–72 ng/mL) and creatine kinase (reference range 19–226 IU/L) level from a patient with *Anncaliia algerae* microsporidiosis, China.

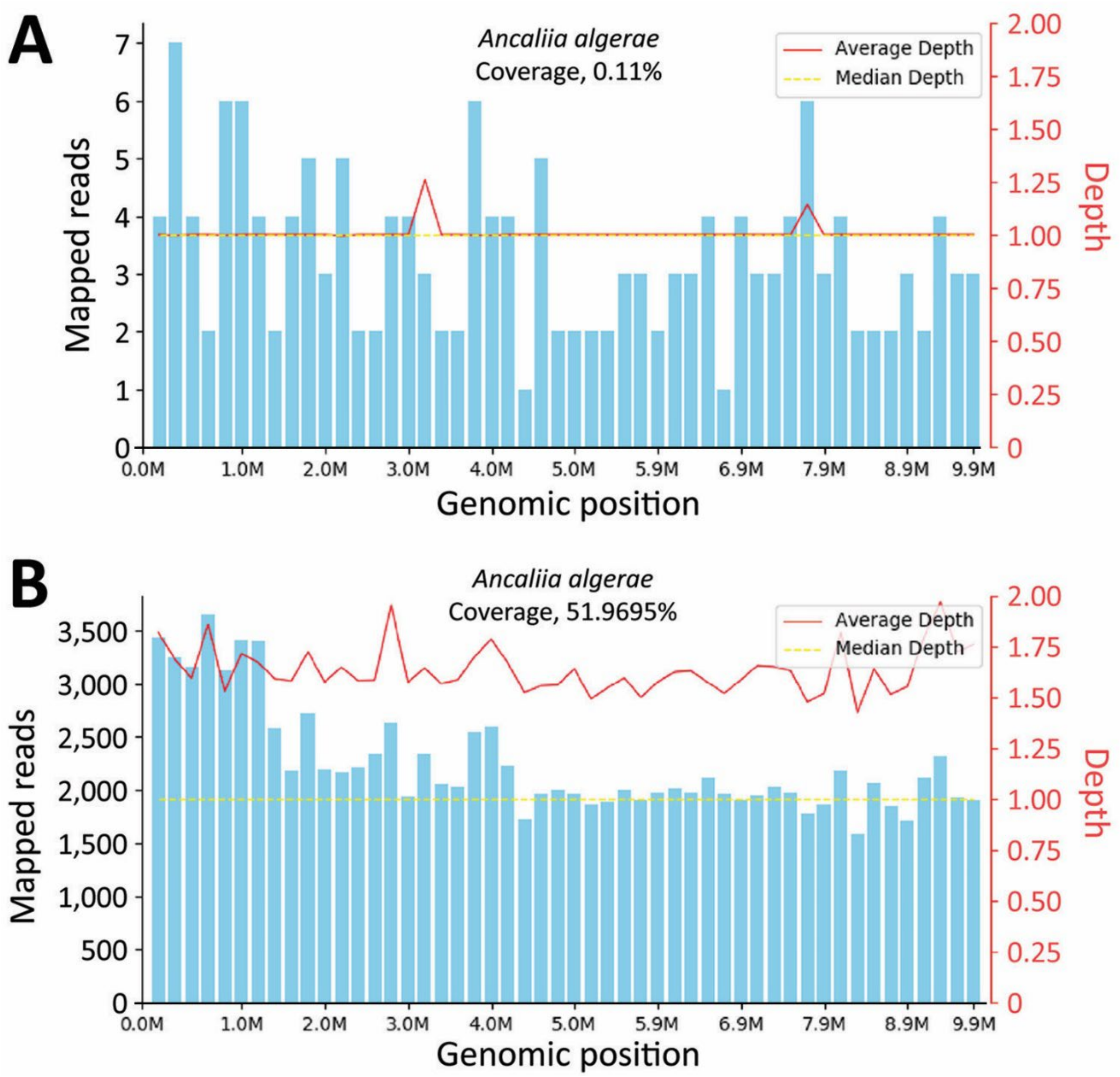

Appendix Figure 2. Results of metagenomic next-generation sequencing of samples from a patient with *Anncaliia algerae* microsporidiosis, China. A) BALF sample; B) left biceps branchii muscle biopsy sample. BALF yielded a total of 127 aligned unique DNA reads mapped to *A. algerae* in the reference database, and the coverage of referenced *A. algerae* genome was 0.11%. Muscle biopsy yielded a total of 65,311 sequence reads mapped to *A. algerae* in the reference database, and the coverage of referenced *A. algerae* genome was 51.97%. The x-axis indicates the nucleotide position in the reference genome, ≈9.9 Mb. BALF, bronchoalveolar lavage fluid.
